# Free Rhodium (II) citrate and rhodium (II) citrate magnetic carriers as potential strategies for breast cancer therapy

**DOI:** 10.1186/1477-3155-9-11

**Published:** 2011-03-28

**Authors:** Marcella LB Carneiro, Eloiza S Nunes, Raphael CA Peixoto, Ricardo GS Oliveira, Luiza HM Lourenço, Izabel CR da Silva, Andreza R Simioni, Antônio C Tedesco, Aparecido R de Souza, Zulmira GM Lacava, Sônia N Báo

**Affiliations:** 1 Instituto de Ciências Biológicas, Universidade de Brasília (UnB), Brazil. 70.919-970; 2 Instituto de Química, Universidade Federal de Goiás (UFG), Brazil.74.001-970; 3 Departamento de Química, Laboratório de Fotobiologia e Fotomedicina, Faculdade de Filosofia, Ciências e Letras de Ribeirão Preto, Universidade de São Paulo, 14040-901, Ribeirão Preto-SP, Brazil

## Abstract

**Background:**

Rhodium (II) citrate (Rh_2_(H_2_cit)_4_) has significant antitumor, cytotoxic, and cytostatic activity on Ehrlich ascite tumor. Although toxic to normal cells, its lower toxicity when compared to carboxylate analogues of rhodium (II) indicates Rh_2_(H_2_cit)_4 _as a promising agent for chemotherapy. Nevertheless, few studies have been performed to explore this potential. Superparamagnetic particles of iron oxide (SPIOs) represent an attractive platform as carriers in drug delivery systems (DDS) because they can present greater specificity to tumor cells than normal cells. Thus, the association between Rh_2_(H_2_cit)_4 _and SPIOs can represent a strategy to enhance the former's therapeutic action. In this work, we report the cytotoxicity of free rhodium (II) citrate (Rh_2_(H_2_cit)_4_) and rhodium (II) citrate-loaded maghemite nanoparticles or magnetoliposomes, used as drug delivery systems, on both normal and carcinoma breast cell cultures.

**Results:**

Treatment with free Rh_2_(H_2_cit)_4 _induced cytotoxicity that was dependent on dose, time, and cell line. The IC_50 _values showed that this effect was more intense on breast normal cells (MCF-10A) than on breast carcinoma cells (MCF-7 and 4T1). However, the treatment with 50 μM Rh_2_(H_2_cit)_4_-loaded maghemite nanoparticles (Magh-Rh_2_(H_2_cit)_4_) and Rh_2_(H_2_cit)_4_-loaded magnetoliposomes (Lip-Magh-Rh_2_(H_2_cit)_4_) induced a higher cytotoxicity on MCF-7 and 4T1 than on MCF-10A (p < 0.05). These treatments enhanced cytotoxicity up to 4.6 times. These cytotoxic effects, induced by free Rh_2_(H_2_cit)_4_, were evidenced by morphological alterations such as nuclear fragmentation, membrane blebbing and phosphatidylserine exposure, reduction of actin filaments, mitochondrial condensation and an increase in number of vacuoles, suggesting that Rh_2_(H_2_cit)_4 _induces cell death by apoptosis.

**Conclusions:**

The treatment with rhodium (II) citrate-loaded maghemite nanoparticles and magnetoliposomes induced more specific cytotoxicity on breast carcinoma cells than on breast normal cells, which is the opposite of the results observed with free Rh_2_(H_2_cit)_4 _treatment. Thus, magnetic nanoparticles represent an attractive platform as carriers in Rh_2_(H_2_cit)_4 _delivery systems, since they can act preferentially in tumor cells. Therefore, these nanopaticulate systems may be explored as a potential tool for chemotherapy drug development.

## Background

Breast carcinoma represents the major cause of death among women worldwide. More than 410,000 deaths are estimated to occur every year, due to its high metastatic capability [1]. This fact demands a continuous development of drugs that may effectively treat breast cancer patients. In point of fact, there is a wide field of research concerning antitumor activity of metal complexes such as platinum [2], ruthenium [3], and rhodium [4]. Among these, rhodium carboxylates are known for their capacity to unpair DNA bases and therefore inhibit DNA synthesis. Their antitumor effect has already been studied on Ehrlich ascites tumor, P388 lymphocytic leukemia, oral carcinoma, L1210 and B16 melanoma, MCa mammary carcinoma and Lewis lung carcinoma [4-6].

**Scheme 1:**
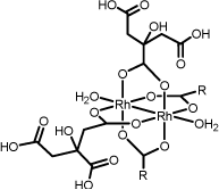
Schematic representation of rhodium (II) citrate showing the possible coordination of the rhodium dimer to the citric acid by the a- and b-carboxyl groups. R groups represent the side chains of citrate ligand

The structure of rhodium (II) citrate (Rh_2_(H_2_cit)_4_), a rhodium carboxylate, is consistent with the familiar dimeric "lantern" structure with bridging carboxylates and a metal-metal bond (Scheme 1). Interestingly, Rh_2_(H_2_cit)_4 _has significant antitumor, cytotoxic, and cytostatic activity on Ehrlich ascites tumor [7]. Although toxic to normal cells, its lower toxicity when compared to carboxylate analogues of rhodium (II) indicates Rh_2_(H_2_cit)_4 _as a promising agent for chemotherapy [4]. Nevertheless, few studies have been performed to explore this potential.

Rh_2_(H_2_cit)_4 _presents uncoordinated functional groups (-COOH and -OH) in its structure. These groups may establish physical or chemical interactions when used in reaction steps with specific molecules or surfaces. Further, these functional groups are chemically similar to bioactive molecules that have been used to functionalize nanostructure materials, such as magnetic nanoparticles, leading to stable colloidal suspensions with excellent biocompatibility and stability [8].

Superparamagnetic particles of iron oxide with appropriate surface functionalization/encapsulation, presented as magnetic fluids or magnetoliposomes, represent an attractive platform as carriers in drug delivery systems (DDS) because they can act specifically in tumor cells [9]. The success of magnetic nanoparticles is mainly due to their high surface area, capacity to pass through the tumor cell membrane and retention to the tumor tissue [10]. In this context, the association between Rh_2_(H_2_cit)_4 _and magnetic nanoparticles, in magnetic fluids or in magnetoliposomes, may work as target-specific drug delivery systems, representing a strategy for enhancement of the therapeutic action of Rh_2_(H_2_cit)_4 _without affecting normal cells.

Some anticancer drugs associated with magnetic nanoparticles such as doxorubicin [11], methotrexate [12], tamoxifen [13], paclitaxel [14], and cisplatin [15] have high potential for chemotherapy. Among the magnetic particles, maghemite (γ-Fe_2_O_3_) is suitable for clinical applications due to its magnetic properties and low toxicity [16]. In this work, we investigated the cytotoxicity induced by (1) free Rh_2_(H_2_cit)_4_, (2) Rh_2_(H_2_cit)_4_-loaded maghemite nanoparticles (Magh-Rh_2_(H_2_cit)_4_) and (3) Rh_2_(H_2_cit)_4_-loaded magnetoliposomes (Lip-Magh-Rh_2_(H_2_cit)_4_) on both normal and carcinoma breast cell cultures.

The association of Rh_2_(H_2_cit)_4 _to magnetic nanoparticles induced specific cytotoxic effect in carcinoma cells. Therefore, we suggest that Magh-Rh_2_(H_2_cit)_4 _and Lip-Magh-Rh_2_(H_2_cit)_4 _may be explored as potential drugs for chemotherapy.

## Results

### • Characterization of rhodium (II) citrate

**Figure 1:**
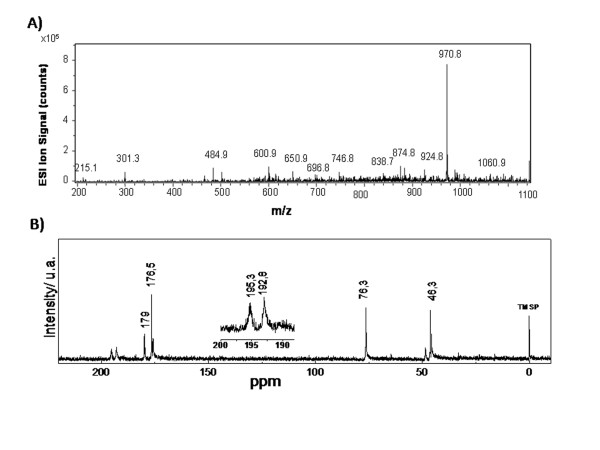
**A) Positive ion ESI-MS spectra of rhodium (II) citrate: [Rh_2_(C_6_H_7_O_7_)_4_+H]^+ ^(*m/z *970,8)**. **Ordinate: relative intensity. B) ^13^C NMR spectra of Rh_2_(H_2_Cit)_4 _complex**. The upper detail shows that the signals of α- and β-carboxyl carbon atoms in the complex (195.3 and 192.8 ppm, respectively) appear shifted.

Elemental analyses of rhodium (II) citrate sample are consistent with the molecular formula [Rh_2_(C_6_H_7_O_7_)_4_(H_2_O)_2_] and suggest, in solid state, the presence of two water molecules in axial position. Thermal studies of the complex showed that the temperature ranged from 25 to140°C, with an estimated mass loss 4.1% (calculated mass loss = 3.6%), which can be accounted for by the loss of the two water molecules. The ESI-MS spectrum of [Rh_2_(C_6_H_7_O_7_)_4_+H]^+ ^(Figure 1A) shows prominent peaks at m/z = 970.8, corresponding to [Rh_2_(C_6_H_7_O_7_)_4 _+ 1H]^+^.

The complex was observed in a ^13^C NMR spectrum (Figure 1B) where the signals of *α*- and *β*-carboxyl carbon atoms in the complex (195.3 and 192.8 ppm, respectively) appear shifted in comparison with those with free ligands (179 and 176.5 ppm, respectively). The shift and split of observed C-O stretching frequencies (from 1740 to 1592 and 1412 cm^-1^) of citric acid in infrared spectra has been used to show the coordination of citric acid to rhodium. The value of Δ(ν_as _CO_2 _- ν_s _CO_2_) = 184 cm^-1 ^observed in the spectrum of rhodium (II) citrate suggests the occurrence of a bridged or chelated bidentate coordination.

The titration of free carboxylic acid groups in the complex provided a ratio of 7.4 ± 0.4 mol H^+ ^by complex mol, indicating a 8:1 stoichiometry predicted by the proposed formula Rh_2_(H_2_cit)_4_.

### • Characterization of Magnetic Nanoparticles and Magnetoliposomes

**Figure 2:**
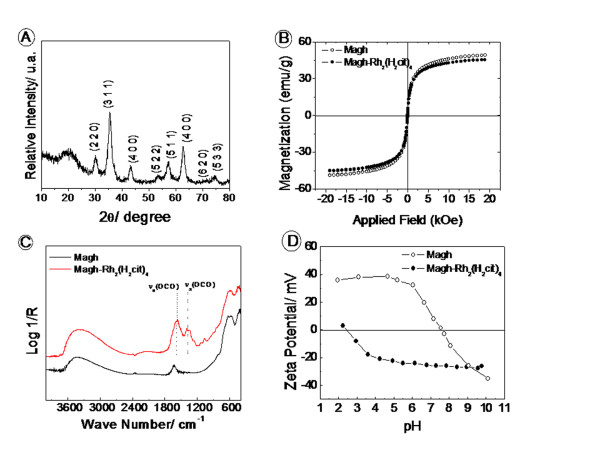
**A) Diffraction pattern for sample Magh**. B) Magnetization curves at 300 K for bare: ○ maghemite (Magh), and ● modified maghemite (Magh-Rh_2_(H_2_cit)_4_). C) Infrared Spectra for ___ Magh and - - - Magh-Rh_2_(H_2_cit)_4_; D) Zeta potential *versus *pH curves for □ ○ □ Magh, and □ ● □ Magh-Rh_2_(H_2_cit)_4_.

SPIOs were obtained in the maghemite (γ-Fe_2_O_3_) phase and presented the characteristic diffraction patterns of inverse spinel structure when compared to reference patterns in the literature [17] for maghemite from the International Center of Diffraction Data [18] (Figure 2A). The molar ratio of Fe^2+^/Fe^3+ ^obtained by elemental analysis was less than 0.015, revealing an efficient oxidation from magnetite to maghemite phase.

The magnetization curves for bare maghemite (Magh) and surface modified maghemite (Magh-Rh_2_(H_2_cit)_4_) are shown in Figure 2B. For both samples, the curves indicate superparamagnetic behavior, since no hysteresis was observed [19,20]. The saturation of magnetization was 48 emug^-1 ^to Magh and 45 emug^-1 ^to Magh-Rh_2_(H_2_cit)_4_.

The surface modification of maghemite nanoparticles was evidenced by infrared spectroscopy and zeta potential measurements. The infrared spectra of functionalized nanoparticles (Figure 2C) show intense absorptions in 1630 and 1564 cm^-1 ^assigned to asymmetrical ν_as_(COO) and symmetrical ν_s_(COO) stretching modes of carboxylate groups [21]. These bands indicate the chemical adsorption of Rh_2_(H_2_cit)_4 _molecules onto the oxide surface [22]. In 1724 cm^-1^, the stretching vibration of carboxylic acid ν(C = O) is observed.

The presence of free acid groups is consistent with obtainment of stable magnetic fluids in physiological pH. The surface Magh-Rh_2_(H_2_cit)_4 _presented a negative zeta potential in a broad range of pH values, and its magnitude in pH 7 was about -35 mV (Figure 2D). The complex and iron oxide content in the sample Magh-Rh_2_(H_2_cit)_4 _were 1.4 mmolL^-1 ^and 0.33 molL^-1^, respectively.

**Figure 3:**
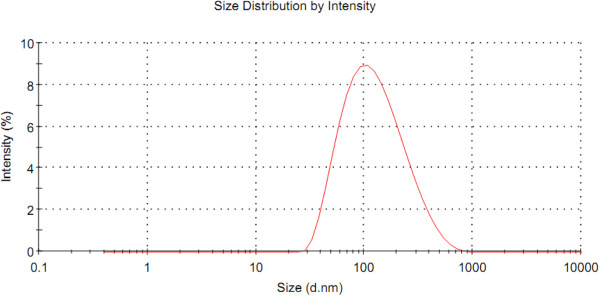
**Size analysis of the magnetoliposomes (1.96 × 10^15 ^iron particles/mL) by laser light scattering**.

The magnetoliposome size presented an average measurement of 101.8 ± 0.1 nm, with polydispersion index lower than 0.22, which corresponded to 98% of the Gaussian distribution (Figure 3).

**Figure 4:**
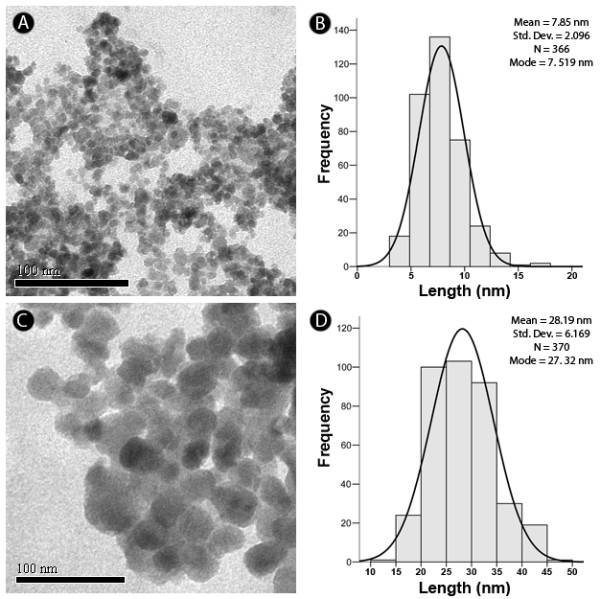
**Morphological characterization and measurement of nanoparticles by transmission electron microscopy**. A) Electron micrograph of maghemite nanoparticles associated with rhodium (II) citrate (Magh-Rh_2_(H_2_cit)_4_, final concentration: 3.12 × 10^13 ^iron particles/mL). B) Histogram of the distribution of the measured diameters of Magh-Rh_2_(H_2_cit)_4_, with a modal diameter mean of 7.85 nm and σ mean = 2.10. C) Electron micrograph of magnetoliposomes associated with rhodium (II) citrate (Lip-Magh-Rh_2_(H_2_cit)_4_, final concentration: 1.25 × 10^13 ^iron particles/mL). D) Histogram of the distribution of diameters of Lip-Magh-Rh_2_(H_2_cit)_4 _showing a mean modal diameter of 28.19 nm and mean σ = 6.17.

TEM micrographs revealed that the maghemite nanoparticles used (Magh-Rh_2_(H_2_cit)_4_) have a spherical shape (Figure 4A) and a modal diameter of 7.85 nm (SD = 2.10) (Figure 4B). In contrast, samples of Lip-Magh-Rh_2_(H_2_cit)_4 _have a rounded shape (Figure 4C) and a modal diameter of 28.19 nm (SD = 6.17) (Figure 4D). Different sized nanoparticles were also observed in the samples, demonstrating their polydispersed distribution.

### • Cytotoxicity of free rhodium (II) citrate

The distribution of cell viability according to the treatment, time, and the evaluated cell line after incubation with free rhodium (II) citrate (Rh_2_(H_2_cit)_4_) is shown in Table 1. A significant difference in the viability of the cells with and without Rh_2_(H_2_cit)_4 _treatment was observed, independently of the cell line and the duration of treatment (p < 0.05). We did not observe cytotoxicity at doses lower than 50 μM Rh_2_(H_2_cit)_4 _(data not shown). All cell lines presented similar cytotoxic effect of 50 μM Rh_2_(H_2_cit)_4 _after 24, 48, and 72 h treatments. However, at doses higher than 200 μM, higher cytotoxicity was observed on breast normal cell line (MCF-10A) than on breast carcinoma cell lines (MCF-7 and 4T1). In general, the cytotoxic effect of Rh_2_(H_2_cit)_4 _was higher after 72 h and after treatments with 500 and 600 μM doses (p < 0.05). Thus, Rh_2_(H_2_cit)_4 _induced a dose and time-dependent viability reduction on the investigated cell lines.

**Table 1:** Distribution of cell viability percentage according to the treatment, cell line and exposure time.

Treatment	Cell line	24 h		48 h		72 h	
**0 (control)**	**MCF-7**	100.00 ± 1.50	A*; a^#^	99.94 ± 1.95	A; a	100.00 ± 1.06	A; a
	**4T1**	100.00 ± 1.21	A; a	100.00 ± 1.46	A; a	100.00 ± 1.34	A; a
	**MCF-10A**	100.00 ± 3.30	A; a	100.00 ± 1.05	A; a	100.00 ± 0.92	A; a

**Rh_2_(H_2_cit)_4 _50 μM**	**MCF-7**	94.96 ± 2.44	A; a	97.48 ± 2.84	A; a	81.19 ± 2.30	B; a
	**4T1**	90.31 ± 1.38	A; a	87.79 ± 2.63	A.B; a	81.42 ± 2.56	B; a
	**MCF-10A**	97.75 ± 3.77	A; a	97.82 ± 1.40	A; a	84.30 ± 2.55	B; a

**Rh_2_(H_2_cit)_4 _200 μM**	**MCF-7**	89.28 ± 2.60	A; a	81.64 ± 2.38	A; a	70.13 ± 2.58	B; a
	**4T1**	79.13 ± 1.44	A; b	73.42 ± 2.17	A.B; a	68.12 ± 3.64	B; a
	**MCF-10A**	61.82 ± 6.54	A; b	44.19 ± 1.60	B; b	30.43 ± 2.69	C; b

**Rh_2_(H_2_cit)_4 _300 μM**	**MCF-7**	85.33 ± 2.14	A; a	73.77 ± 2.58	B; a	54.14 ± 2.47	C; a
	**4T1**	73.95 ± 2.54	A; a	61.77 ± 1.47	B; b	47.79 ± 4.11	C; a
	**MCF-10A**	39.41 ± 7.47	A; b	23.81 ± 0.74	B; c	12.78 ± 0.92	C; b

**Rh_2_(H_2_cit)_4 _500 μM**	**MCF-7**	50.08 ± 2.49	A; a	25.29 ± 3.46	B.C; a	30.39 ± 3.47	C; a
	**4T1**	46.14 ± 3.49	A; a	30.66 ± 1.22	B; a	26.07 ± 2.75	B; a
	**MCF-10A**	25.85 ± 6.46	A; b	11.62 ± 1.17	A.B; b	5.46 ± 0.46	C; b

**Rh_2_(H_2_cit)_4 _600 μM**	**MCF-7**	28.71 ± 3.90	A; a	16.86 ± 1.77	B; a	12.16 ± 1.93	B; a
	**4T1**	29.87 ± 3.67	A; a	15.86 ± 0.57	B; a	9.97 ± 1.49	B; a
	**MCF-10A**	13.34 ± 2.43	A; b	10.26 ± 1.27	A; b	4.76 ± 0.39	B; b

**DMSO (0.85%)**	**MCF-7**	90.51 ± 5.9	A; a	90.93 ± 1.7	A; a	96.4 ± 1.4	A; a
	**4T1**	106.2 ± 1.3	A; b	100.6 ± 2.97	A; a	43.07 ± 8.2	B; b
	**MCF-10A**	148.1 ± 6.8	A; c	82.45 ± 2.3	B; a	63.35 ± 2.2	C; c

**Paclitaxel 50 μM**	**MCF-7**	70.07 ± 0.4	A; a	55.93 ± 1.6	B; a	18.92 ± 4.3	C; a
	**4T1**	68.31 ± 1.2	A; a	30.12 ± 0.7	B; b	21.51 ± 1.4	C; a
	**MCF-10A**	80.17 ± 6.7	A; c	33.52 ± 1.09	B; b	20.95 ± 1.1	C; a

The data represent the mean ± SE (mean standard error) of three independent experiments in triplicates. * Different capital letters denote statistical difference between viability in the different times (rows) for a given cell line (breast cancer cells MCF-7. 4T1 or normal cells MCF-10A) under the same treatment (p <0.05). # Different tiny letters indicate mean statistical difference between the viability of different cell lines (columns) for a given time (24. 48 or 72 hours) (p <0.05).

Paclitaxel (50 μM), used as positive control, induced a more intense cytotoxic effect after 72 h in the three cell lines than Rh_2_(H_2_cit)_4_. Treatments with DMSO caused no significant cytotoxicity to the three cell lines studied after 24 and 48 h treatments. Nevertheless, after 72 h, DMSO demonstrated a higher cytotoxicity to 4T1 and MCF-10A cells lines than to MCF-7 line. Since the cells studied showed sensitivity to paclitaxel our experimental models were validated (Table 1).

The IC_50 _values of the treatments with Rh_2_(H_2_cit)_4 _in MCF-7, 4T1, and MCF-10A cells are shown in Table 2. The results confirmed that the cytotoxicity of the treatment with the complex is dependent on dose, time, and cell line. The IC_50 _values for human carcinoma (MCF-7) and mouse carcinoma (4T1) cell lines were relatively similar. Likewise, normal cell lines (MCF-10A) were more sensitive to treatment with Rh_2_(H_2_cit)_4 _(Table 2).

**Table 2:** Distribution of the IC_50 _values and their respective confidence intervals (95%) in MCF-7, 4T1, and MCF-10A cell lines after treatment with free rhodium (II) citrate (Rh_2_(H_2_cit)_4_).

	IC_50 _(IC 95%)					
	
Cell lines	24 hours		48 hours		72 hours	
**MCF-7**	483 μM	(459,2 a 507 μM)	376 μM	(356,2 a 396,1 μM)	294 μM	(259,9 a 332,5 μM)
**4T1**	440 μM	(407,3 a 475 μM)	337 μM	(317,3 a 357,8 μM)	271 μM	(241,4 a 303,9 μM)
**MCF-10A**	250 μM	(211,1 a 295,2 μM)	181 μM	(172,3 a 190,8 μM)	123 μM	(114,7 a 132,7 μM)

These data refers from viability of cells after treatment with Rh_2_(H_2_cit)_4 _(50-600 μM) for 24, 48 and 72 hours.

### • Analysis of morphological and structural alterations on MCF-7 and 4T1 cell lines

**Figure 5:**
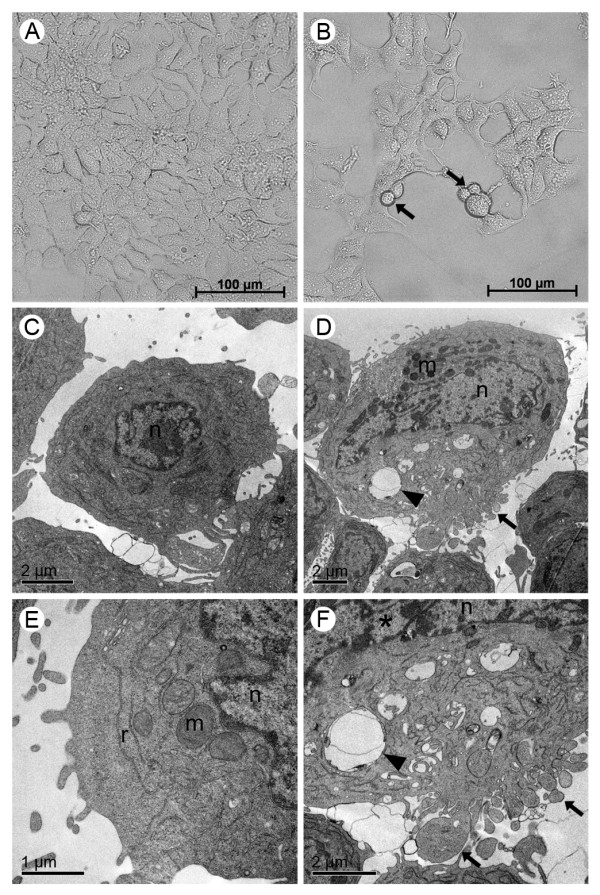
**Morphological and structural changes induced by rhodium (II) citrate (Rh_2_(H_2_cit)_4_) in MCF-7 breast carcinoma cell line after 48 hours of treatment**. Cells were incubated with 500 μM Rh_2_(H_2_cit)_4 _for 48 hours and examined by phase contrast microscopy (A, B) and transmission electron microscopy (C-F). (A, C and E) control (cells without treatment); (B, D and F) cells treated with 500 μM of Rh_2_(H_2_cit)_4_. Differences were observed in cell morphology, vacuole amount and mitochondrial condensation between untreated cells (A, C and E) and Rh_2_(H_2_cit)_4 _treated cells (B, D and F). Legends: blebbing (arrows), vacuoles (arrow heads), nucleus (n), mitochondria (m), condensed chromatin (*).

**Figure 6:**
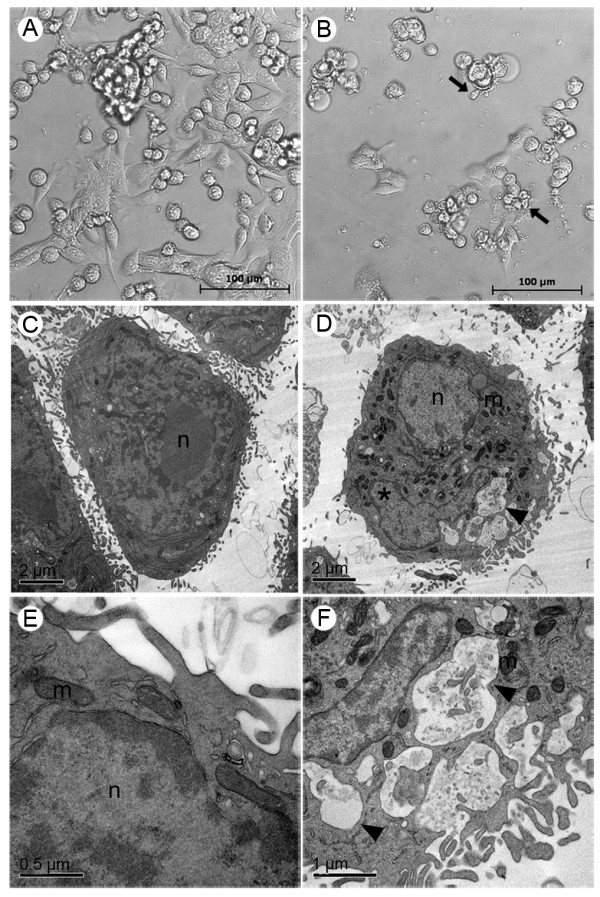
**Morphological and structural changes induced by rhodium (II) citrate (Rh_2_(H_2_cit)_4_) in 4T1 breast carcinoma cell line after 48 hours of treatment**. Cells were incubated with 500 μM Rh_2_(H_2_cit)_4 _for 48 hours and examined by phase contrast microscopy (A, B) and transmission electron microscopy (C-F). (A, C and E) control (cells without treatment); (B, D and F) cells treated with 500 μM of Rh_2_(H_2_cit)_4_. Differences were observed in cell morphology, vacuole amount and mitochondrial condensation between untreated cells (A, C and E) and Rh_2_(H_2_cit)_4 _treated cells (B, D and F). Legends: blebbing (arrows), vacuoles (arrow heads), nucleus (n), mitochondria (m), condensed chromatin (*).

MCF-7 cells have predominantly fusiform morphology (Figure 5A), while 4T1 cells presented both spindle and rounded cells forming clusters, characteristic of this these types of tumor cells (Figure 6A). Nevertheless, both MCF-7 and 4T1 cells became more rounded and with blebbing after treatment with 500 μM Rh_2_(H_2_cit)_4 _for 48 h. After this treatment smaller confluence and reduced cell size were also observed when 4T1 and MCF-7 control cells were compared. Furthermore, this effect was more pronounced in the 4T1 cell line (Figure 5A, B and 6A, B). No morphological alterations were observed in MCF-7 and 4T1 untreated cells (control), according to the images taken by the phase contrast microscope (Figure 5A and 6A).

Ultrastructural details of MCF-7 and 4T1 cell morphology, after treatment with 500 μM Rh_2_(H_2_cit)_4_, are shown in Figure 5D, F and 6D, F, respectively. After this treatment, several morphological alterations were observed, such as the presence of blebbing, the segregation of condensed chromatin to nuclear periphery and the remarkable presence of vacuoles and condensed mitochondria when compared to the MCF-7 and 4T1 control cells (Figure 5C-F and 6C-F), respectively. These morphological changes can be related to the apoptotic events.

### • Phosphatidylserine exposition on breast carcinoma cells

**Figure 7:**
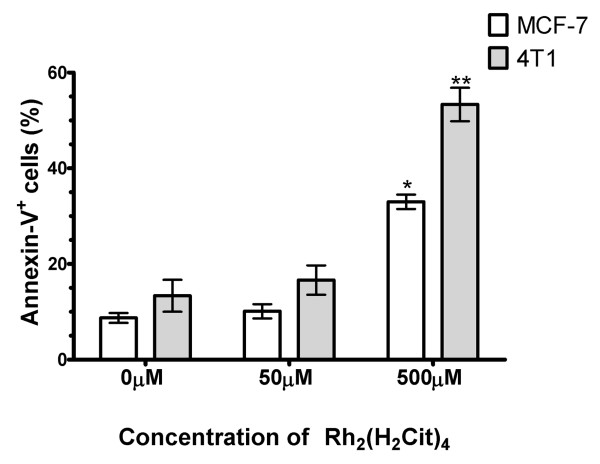
**Phosphatidylserine exposure induced by rhodium (II) citrate (Rh_2_(H_2_cit)_4_) in breast carcinoma cells (lines 4T1 and MCF-7) after 48 hours of treatment**. Cells were stained with annexin V-FITC (fluorescein-5-isothiocyanate) and PI (propidium iodide) and analyzed by flow cytometry. The percentage of annexin positive cells represents the cells with exposed phosphatidylserine. Data were normalized with the control (cells without treatment) and expressed as percentage of the mean ± SE of three experiments that were independently performed in triplicate. One or two asterisks (* and **) indicate statistical differences between control and cells treated in MCF-7 and 4T1 cell lines, respectively (p < 0.001).

In Figure 7 the percentage of cells that were positively stained for annexin V-FITC is represented. After 500 μM Rh_2_(H_2_cit)_4 _treatment, the annexin-V^+ ^cell number (%) was significantly higher than that of the control in both cell lines (p < 0.05). After this treatment, there was a 25% and a 38% increase of annexin-V^+ ^cell number in MCF-7 and 4T1, respectively (p < 0.05), thus revealing that the 4T1 cell line was more sensitive to treatment with Rh_2_(H_2_cit)_4 _(500 μM). No difference in the percentage of annexin-V^+ ^cell number was observed in relation to untreated cells (control) and 50 μM Rh_2_(H_2_cit)_4 _treated cells, in both cell lines (p < 0.05).

### • Analysis of nuclear fragmentation and actin alterations

**Figure 8:**
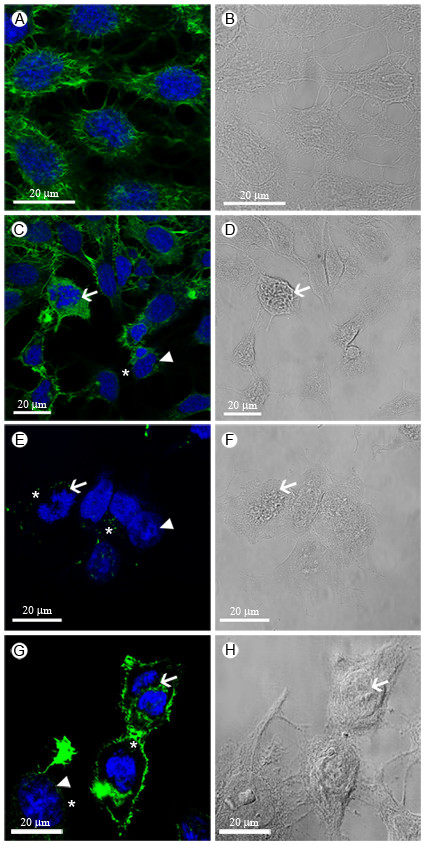
**Nuclear fragmentation and reduction of actin filaments in MCF-7 breast carcinoma cells 48 hours after treatment**. Cells were stained with DAPI (4',6-diamidino-2-fenilindol) to visualize the nucleus (in blue) and with Phalloidine-Alexa Fluor 488 to visualize actin (in green). (A, B) control (cells without treatment); (C, D) cells treated with 50 μM and (E, F) with 500 μM of Rh_2_(H_2_cit)_4_; (G, H) cells treated with 10 nM paclitaxel for 2 h. Arrows and arrow heads indicate nuclear fragmentation and chromatin condensation, respectively. Phase-contrast images are presented for validation of fluorescence (Figure 8B, D, F, H).

MCF-7 cells without treatment (control) showed organized spread actin in the cytoplasm and interactions between surrounding cells through membrane projections supported by actin (Figure 8A). After treatment with 50 μM Rh_2_(H_2_cit)_4_, slight nuclear condensation and reduction of actin filaments were observed (Figure 8C). Nevertheless, a noticeable reduction in actin and increased nuclear condensation were observed after treatment with 500 μM (Figure 8E). In general, the cells treated with Rh_2_(H_2_cit)_4 _showed a loss of cytoplasmic projections when compared to the control cells (Figure 8A, C and 8E). Furthermore, the cells treated with paclitaxel (50 μM) showed nuclear condensation and fragmentation and a lower amount of actin cytoskeleton, similar to those treated with Rh_2_(H_2_cit)_4 _(Figure 8G). Phase contrast images were shown to validate DAPI and phalloidin-Alexa Fluor 488 staining for each experimental group (Figure 8B, D, F and 8H).

### • Cytotoxicity of rhodium (II) citrate-loaded magnetic nanoparticles

**Figure 9:**
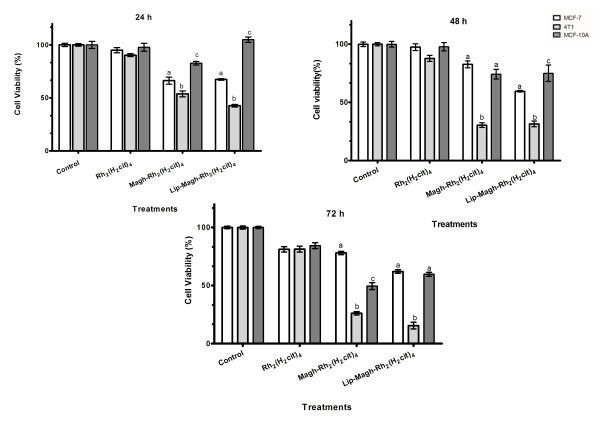
**Cytotoxic effect of maghemite nanoparticles associated with rhodium (II) citrate (Magh-Rh_2_(H_2_cit)_4_) and magnetoliposomes (Lip-Magh-Rh_2_(H_2_cit)_4_) in breast carcinoma cell lines (MCF-7 and 4T1) and breast normal cell line (MCF-10A)**. Cells were incubated with free rhodium (II) citrate (Rh_2_(H_2_cit)_4_), Magh-Rh_2_(H_2_cit)_4 _(final concentration: 3 × 10^15 ^iron particles/mL and 23 mM of iron) or Lip-Magh-Rh_2_(H_2_cit)_4 _(final concentration: 12.5 × 10^15 ^iron particles/mL and 94.5 mM of iron) for 24, 48 and 72 h. In all treatments the concentration of Rh_2_(H_2_cit)_4 _used was 50 μM. Data were normalized with the control (cells without treatment) and expressed as mean ± standard error of two independent experiments performed in triplicates. Different letters indicate statistical difference within each treatment (p < 0.05).

MCF-7, 4T1, and MCF-10A cell viabilities were similar after treatment with 50 μM of free Rh_2_(H_2_cit)_4_, independent of the treatment duration (Figure 9). Nevertheless, treatment with 50 μM Rh_2_(H_2_cit)_4_-loaded maghemite nanoparticles (Magh-Rh_2_(H_2_cit)_4_) and Rh_2_(H_2_cit)_4_-loaded magnetoliposomes (Lip-Magh-Rh_2_(H_2_cit)_4_) induced a significant decrease, mainly in MCF-7 and 4T1 breast carcinoma cell viability (p < 0.05). This effect was more evident in 4T1 cells, which showed a fall in viability of 46% (± 2.7), 69% (± 2), and 74% (± 1.4) after Magh-Rh_2_(H_2_cit)_4 _treatment for 24, 48, and 72 h, respectively. Within the same time frame, the Lip-Magh-Rh_2_(H_2_cit)_4 _treatment decreased 4T1 cell viability by 57% (± 1.3), 68% (± 2.4), and 84% (± 2.9) after 24, 48 and 72 h treatments, respectively (Figure 9). In contrast, the same dose of free Rh_2_(H_2_cit)_4 _reduced cell viability by about 10% (± 1.4), 12% (± 2.6), and 18% (± 2.6), after 24, 48 and 72 h treatments, respectively (p < 0.05). However, 72 h of Magh-Rh_2_(H_2_cit)_4 _and Lip-Magh-Rh_2_(H_2_cit)_4 _treatments on 4T1 cells induced a decrease in cell viability of respectively 74% (± 1.4) and 84% (± 2.9) against 18% (± 2.6) presented by the free drug at the same concentration. Thus, Magh-Rh_2_(H_2_cit)_4 _and Lip-Magh-Rh_2_(H_2_cit)_4 _treatments showed enhanced Rh_2_(H_2_cit)_4 _potency of up to 3.9 and 4.6 times, respectively.

Longer treatments enhanced the cytotoxicity of both Magh-Rh_2_(H_2_cit)_4 _and Lip-Magh-Rh_2_(H_2_cit)_4 _(Figure 9). After 24 h of treatment with Magh-Rh_2_(H_2_cit)_4 _and Lip-Magh-Rh_2_(H_2_cit)_4_, a differential cytotoxicity was observed among the three cell lines. This effect was more pronounced in 4T1 and MCF-7 cells. Further, we observed that Lip-Magh-Rh_2_(H_2_cit)_4 _treatment was more cytotoxic than Magh-Rh_2_(H_2_cit)_4 _to MCF-7 cell line (p < 0.05). A higher cytotoxicity was noticed in MCF-10A 72 h after the Magh-Rh_2_(H_2_cit)_4 _treatment, but this did not happen with the Lip-Magh-Rh_2_(H_2_cit)_4 _treatment. It is noteworthy that in all time windows and all tested cell lines there was no difference in the viability of the control cells (p < 0.05) (Figure 9).

The cells treated with maghemite nanoparticles without rhodium (II) citrate (Magh) showed no reduction in viability after any treatment duration; however, viability reduction was observed after 72 h treatment with Lip-Magh (data not shown).

## Discussion

In this work, the rhodium (II) citrate was isolated from the aqueous solution as powder and not as a single crystal. Due to this fact the complete structure determination cannot be resolved. However, the elemental analysis, ^13^C NMR, IR, UV/Visible data enable us to predict that the compound structure was similar to the previously studied rhodium (II) carboxylates [23]. In the ^13^C NMR spectrum (Figure 1B), the signals of *α*- and *β*-carboxyl carbon atoms in the complex appear shifted in comparison with those for the free ligand, showing that the citrate anion is coordinated through these two carboxyl groups. In the carbinol carbon atoms, however, only a small shift is observed, indicating that there is no participation of this group in coordination [24,25]. Evidence of the coordination of citric acid ligand to rhodium though its carboxyl group was also obtained by infrared spectra, and it was similar to that reported by Najjar and co-workers for rhodium (II) citrate [26]. The coordination by the two different carboxyl groups suggests the formation of five isomeric structures; however, for the development of this work these hypothetic isomers were not separated.

The crystalline structure of magnetic nanoparticles could be confirmed by X-ray difractometry as maghemite phase. According to Magh and Magh-Rh_2_(H_2_cit)_4 _magnetization curves profile (Figure 2B), the nanoparticles present superparamagnetic behavior at room temperature and saturation magnetization close to values already published in the literature for 7 nm maghemite. The effect of the complex on the particle's surface to saturation magnetization is negligible [27].

Surface functionalization of SPIO with rhodium (II) citrate produced deep changes in the nanoparticles' physical-chemical properties. These changes were evidenced by infrared spectroscopy and zeta potential measurements, as well as by saturation of magnetization. The infrared spectra of Magh-Rh_2_(H_2_cit)_4 _(Figure 2C) showed intense absorptions assigned to asymmetrical ν_as_(COO) and symmetrical ν_s_(COO) stretching modes of carboxylate groups [21], indicating the chemical adsorption of Rh_2_(H_2_cit)_4 _molecules into the oxide surface [22]. Zeta potential *versus *pH measurements indicated an isoelectric point (iep) at about pH 3. The zeta potential becomes negative in the range of pH above 3 and its magnitude at pH 7 is about -38 mV. This zeta potential value shows that the particles are negatively charged and indicates an efficient electrostatic stabilization.

It is well known that the magnetic properties of nanomaterials are dependent on their size. Particles smaller than 10 nm, besides having high magnetic applicability, are also ideal to avoid recognition by the mononuclear phagocyte system and, thus, stay longer in the bloodstream [16]. Considering the particle size, Magh-Rh_2_(h_2_cit)_4 _has potential for applications in the biological system as it presents a modal diameter of 7.5 nm. Moreover, considering the magnetoliposome size, as determined by zetasizer equipment (Figure 3), we could conclude that the small lipid bilayer vesicle will increase the interaction of the active compounds with cells as a normal behavior of other liposomal drug delivery systems (DDS) of similar size carrying similar nanoparticles to the cellular target [28].

In our *in vitro *study, we observed that cell lines MCF-7, 4T1, and MCF-10A exhibited cytotoxicity when treated with Rh_2_(H_2_cit)_4_. It is reported that others carboxylates such as acetate, butyrate, and propionate of rhodium, in association with isonicotinic acid, also induces cytotoxicity in tumor cells (K562 leukemia cell line) [29]. We also observed that Rh_2_(H_2_cit)_4 _cytotoxicity was dose and time dependent. High concentrations of Rh_2_(H_2_cit)_4 _(up to 200 μM) were seen to induce greater cytotoxicity after longer treatments (72 hours). Furthermore, it was also demonstrated that its cytotoxic effect differed between breast normal (MCF-10A) and breast carcinoma (4T1 and MCF-7) cell lines, being more pronounced in breast normal cells (Table 1 and 2). Our data are, therefore, in agreement with a number of other preliminary studies. For instance, preliminary studies showed that rhodium (II) citrate induces a higher cytotoxicity, with increasing dose and duration of treatment, on breast carcinoma cells (Ehrlich) and on carcinoma (Y-1) and normal adrenocortical cells (AR-1(6)) [7]. Similarly, it was also reported that other rhodium carboxylates such as acetate, methoxyacetate, propionate, and butyrate inhibited the proliferation of leukemia cells (L1210), inducing cytotoxic effects in a dose and a time-dependent manner [30].

Several studies reported promising antitumor activities of rhodium carboxylates in mouse bearing Ehrlich breast carcinoma, but their clinical use has been limited because they showed toxicity in normal cells [4,31]. In our study, Rh_2_(H_2_cit)_4 _was also cytotoxic to *in vitro *normal cells. The IC_50 _values (Table 2) showed that Rh_2_(H_2_cit)_4 _cytotoxic effect was more intense on breast normal cells (MCF-10A) than on breast carcinoma cells (MCF-7 and 4T1). However, according to the IC_50 _values (Table 2), we demonstrated that rhodium (II) citrate is less toxic to normal cells than are members of the lipophilic complex, such as propionate, butyrate, and acetate of rhodium [30]. Therefore, this complex may have a higher chemotherapeutic potential in relation to other carboxylates. The distinctness of cytotoxicity among lipophilicity por hydrophilicity carboxylates could be explained by the differences among their properties, such as chain length and hydrophilicity of parts of the molecules [4].

The cytotoxic activity of some rhodium carboxylates is given by their ability to bind covalently to DNA bases, unpairing them, and subsequently inhibiting DNA replication and transcription [5,7]. It was reported that rhodium carboxylates establish adducts through their axial ligands with electron donor atoms, preferably N, S, O, and P, from molecules such as adenine, cysteine, and RNase A [32]. Moreover, enzymes with free thiol groups (-SH) are known to interact irreversibly with these metal complexes [30]. This interaction could explain the inactivation of some essential DNA replication enzymes which result in their damage. Thus, Rh_2_(H_2_cit)_4 _is toxic to both normal and carcinoma cells since they need DNA replication and transcription to survive. Zyngier and colleagues [7] demonstrated that Rh_2_(H_2_cit)_4 _inhibited DNA synthesis of breast carcinoma (Ehrlich), and also of carcinoma (Y-1) and, normal adrenocortical cells (AR-1(6)). We observed fragmentation nucleus induced by Rh_2_(H_2_cit)_4 _(Figure 5D and 6D, Figure 8C and 8E). These observations suggest that Rh_2_(H_2_cit)_4 _not only induces DNA fragmentation on MCF-7 and 4T1 cells, but may also prevent their DNA synthesis.

According to our TEM observations, the MCF-7 and 4T1 cells exhibited condensed mitochondria after Rh_2_(H_2_cit)_4 _treatment (Figure 5D, F and 5F), indicating that this organelle is somehow affected by the complex. This condensed mitochondria phenotype can be associated with a drop in the mitochondrial membrane potential related to the cell death process [33].

We observed that Rh_2_(H_2_cit)_4 _induced an increase in the number of vacuoles compared to the untreated cells, as shown in TEM (Figure 5 and 6). It can indicate a degradation pathway related to the response to metabolic stress or microenvironmental conditions to ensure energy balance. Moreover, this increase has been implicated in the cell death process [34,35].

After 48 h of treatment with 500 μM Rh_2_(H_2_cit)_4, _an increase of annexin-V^+ ^breast carcinoma cells was observed (Figure 7). The presence of annexin-V^+ ^in cells is related to apoptotic events, since it indicates the exposure of phosphatidylserine outside the inner membrane. The actin analysis performed by confocal microscopy showed a dose-dependent disassembly of the actin cytoskeleton after Rh_2_(H_2_cit)_4 _treatment in the MCF-7 cell (Figure 8). Furthermore, there was a notable reduction in intercellular communication, possibly caused by changes in the actin cytoskeleton (Figure 8). This structure is an important target for many antitumor drugs since it plays a crucial role in maintaining cell morphology, mitosis, signaling regulation for cell survival, and cell motility [36-38]. We demonstrated that the reduction of actin after Rh_2_(H_2_cit)_4 _treatment (500 μM) is intrinsically related to the higher cytotoxicity of this complex in MCF-7 cells (Table 1 and Figure 8).

In summary, Rh_2_(H_2_cit)_4 _induces alterations in the treated cells that are related to the apoptosis process, such as nuclear fragmentation, blebbing, disassembly of the actin cytoskeleton, and phosphatidylserine exposure in the plasma membrane. These features suggest that Rh_2_(H_2_cit)_4 _has potential as an efficient chemotherapic agent since targeting of chemotherapeutic agents is related to its capacity to induce apoptosis.

In order to reduce the toxicity of Rh_2_(H_2_cit)_4 _for normal cells while enhancing the efficacy in carcinoma therapy, we proposed its association with magnetic nanoparticles. Doses of 50 μM of Rh_2_(H_2_cit)_4_-loaded to maghemite nanoparticles and to magnetoliposomes were more cytotoxic than the equimolar dose of free Rh_2_(H_2_cit)_4_. Besides, the treatment with 50 μM of Magh-Rh_2_(H_2_cit)_4 _induced cytotoxicity similar to a tenfold dose of the free complex on carcinoma cells. In addition, the Magh-Rh_2_(H_2_cit)_4 _and Lip-Magh-Rh_2_(H_2_cit)_4 _induced time-dependent cytotoxic effect like those of free Rh_2_(H_2_cit)_4_. After 72 h, for example, Magh-Rh_2_(H_2_cit)_4 _and Lip-Magh-Rh_2_(H_2_cit)_4 _treatments enhanced cytotoxicity potency up to 3.9 and 4.6 times, respectively. More importantly, MCF-7 and 4T1 carcinoma breast cells were more susceptible to Magh-Rh_2_(H_2_cit)_4 _and Lip-Magh-Rh_2_(H_2_cit)_4 _treatments than MCF-10A normal breast cells, differently from what is observed with free Rh_2_(H_2_cit)_4 _(Table 2 and Figure 9).

Carcinoma and normal cells present different metabolism in relation to iron uptake. The metabolism of breast carcinoma cells, for example, is faster than in normal cells. Consequently, carcinoma cells require larger amounts of micronutrients, particularly iron, which can be evidenced by the presence of more transferrin receptors in these [39]. In this way, an increased iron uptake by tumor cells could result in a selective uptake and a higher retention of Magh-Rh_2_(H_2_cit)_4 _and Lip-Magh-Rh_2_(H_2_cit)_4 _in relation to free Rh_2_(H_2_cit)_4 _complex. Additionally, magnetic nanoparticle uptake by carcinoma cells may also be associated with the amino group's coverage of nanoparticles [40]. The literature reports that free thiol groups (-SH) interact with the rhodium carboxylates, which are rich in carboxylic groups [30]. Therefore, the carboxylic groups present in Magh-Rh_2_(H_2_cit)_4 _citrate molecules could improve the transport of nanoparticles through the cell membrane via the proteic thiol groups.

Although rhodium (II) citrate-coated maghemite nanoparticles seem not to have been described before, the association of rhodium complex with polymeric microspheres of hydroxy-propyl-cyclodextrin [41] and with cyclodextrins from hydroxyapatite has been reported [42]. These associations were shown to represent a promising alternative in the minimization of the nonspecific toxicity of these agents, mainly because they increase the efficiency of encapsulation and the duration of rhodium (II) citrate release. Our study demonstrated that the composition of maghemite nanoparticles coated with citrate or rhodium (II) citrate was appropriate for its application as a drug delivery system. Coating with the citrate molecule was able to stabilize our magnetic nanoparticles and also was not toxic to the investigated cells (data not shown). Citrate-functionalized-maghemite has been attested as providing successful nanoparticles in the production of biocompatible and stable magnetic fluids [43,44]. Furthermore, citrate-functionalized-maghemite was also shown to be internalized by *in vitro *human melanoma cells (SKMEL 37) with no significant cytotoxicity even when cultivated for 72 h [45].

We demonstrated that Magh-Rh_2_(H_2_cit)_4 _and Lip-Magh-Rh_2_(H_2_cit)_4 _compositions reduced more efficiently the viability of MCF-7 and 4T1 breast carcinoma cells than the free Rh_2_(H_2_cit)_4 _treatment. Furthermore, it is important to emphasize that the cytotoxicity induced by both Magh-Rh_2_(H_2_cit)_4 _and Lip-Magh-Rh_2_(H_2_cit)_4 _was greater in tumor cells than normal ones, since no cytotoxicity was observed after treatment with Magh. In addition, if these nanosystems were associated to target molecules for breast carcinoma cells such as folic acid, for instance, their potential for selective uptake would be even higher [46]. Thus, Magh-Rh_2_(H_2_cit)_4 _and Lip-Magh-Rh_2_(H_2_cit)_4 _have much potential for application in drug delivery systems, and they should be considered as a platform to enhance Rh_2_(H_2_cit)_4 _cytotoxicity, specifically in breast carcinoma.

## Conclusions

We showed that Rh_2_(H_2_cit)_4 _induces significant cytotoxic effects, especially after longer treatments and at higher concentrations. These effects were related to several structural and morphological alterations, probably coming from cell death by apoptosis and autophagy. Further, higher cytotoxicity in the MCF-10A breast normal cell line was noted than in the 4T1 and MCF-7 breast cancer cell lines. Nonetheless, the Magh-Rh_2_(H_2_cit)_4 _and Lip-Magh-Rh_2_(H_2_cit)_4 _treatments were more selective to breast cancer cells with up to 4.6 times enhanced potency in comparison to the free Rh_2_(H_2_cit)_4_. Therefore, we suggest that Magh-Rh_2_(H_2_cit)_4 _and Lip-Magh-Rh_2_(H_2_cit)_4 _should be considered a suitable and effective platform for drug delivery systems that operate more specifically in tumor cells.

## Methods

### Materials

All solvents and reagents related to the synthesis of Rh_2_(H_2_cit)_4 _and Magh-Rh_2_(H_2_cit)_4 _are of analytical grade and were used without further purification: iron(II) chloride tetrahydrate (Acros); iron (III) chloride hexahydrate (Ecibra); hydrated rhodium (III) chloride (Sigma-Aldrich); citric acid (Vetec), and sodium hydroxide (FMaia). The rhodium(II) trifluoroacetate, [Rh_2_(tfa)_4_], was prepared following a previously reported procedure [47].

### • Characterization of Rhodium Compounds

Infrared spectra were recorded using KBr pellets on a Bomem BM100 FT-IR spectrometer in the 4000-500 cm^-1 ^region. Elemental analyses were carried out on a Perkin-Elmer 2400 analyzer. Rhodium concentrations were measured in Spectro Ciros CCD ICP-AES spectrometer. The samples were digested with concentrated HCl in an aqueous solution. Electronic spectra were recorded in the 800-200 nm range on Beckman DU70 spectrometer in water solution. The ^13^C NMR spectra (carbon-13 nuclear magnetic resonance spectroscopy) were obtained at room temperature in D_2_O using a Bruker Avance III 500 spectrometer, operating at a frequency of 125.75 MHz. The ^13^C chemical shifts were measured relative to TMS (tetramethylsilane) measurements. TGA (thermogravimetric analysis) was performed at a heating rate of 10°C min^-1 ^in the temperature range of 25-1000°C, under nitrogen flow of 10 mL min^-1 ^using a Shimadzu DTG-60 instrument and standard aluminum crucible. The ESI mass spectra (Electrospray ionisation-mass spectrometry) were acquired using a Bruker Daltonics Esquire 3000 Plus mass spectrometer in capillary exit voltage set at 4 kV and the desolvation chamber temperature was set to 280°C. Potentiometric titration of an aqueous solution of Rh_2_(H_2_cit)_4 _0.0051 molL^-1 ^was performed in triplicate using a 0.046 molL^-1 ^NaOH solution as titrant.

### • Characterization of Magnetic Nanoparticles

X-ray powder diffraction (XRD) data were collected by a XRD-6000 diffractometer. The magnetization of the iron oxide nanoparticles was measured at room temperature using a vibrating-sample magnetometer (EV9-VSM AdMagnets). The iron concentration in the fluids was determined by the method of *o*-phenanthroline [48]. Solution absorbances were measured at 512 nm in a Hitachi U1100. Zeta potential was obtained from electrophoretic mobility (em) measurements performed by phase analysis light scattering using ZetaSizer Nano ZS ZEN3600 (Malvern, UK) equipment. The mean hydrodynamic particle size of Magh-Rh_2_(H_2_cit)_4 _was determined in water by dynamic laser light scattering (DLS) and the correlation functions were evaluated by cumulant analysis. Maghemite nanoparticles were dispersed in an electrolyte (0.005 molL^-1 ^NaCl) solution to get a 0.05 molL^-1 ^iron content.

Moreover, to determine the nanoparticles' shape and size by transmission electron microscopy (TEM) an aliquot (10 μL) of synthesized (Magh-Rh_2_(H_2_cit)_4_) (0.2%) and Lip-Magh-Rh_2_(H_2_cit)_4 _(0.4%) was deposited on a copper grid (300 mesh), previously covered with Formvar (0.7%), and dried at room temperature. It was then observed under transmission electron microscopy (TEM, JEOL 1011, 100kV) and the images were captured by a Gatan Ultrascan camera. Nanoparticles (n = 370) were measured by Image Pro-Plus 5.1 software and data were adjusted by log normal distribution to obtain the modal diameter.

### • Synthesis of the Rhodium (II) Citrate Complex, Rh_2_(H_2_cit)_4_

Firstly, an aqueous solution of rhodium (II) trifluoroacetate (*c.a*. 1 mmol) was slowly added to a solution of citric acid (*c.a*.10 mmol) in water under stirring and heated to 70°C. The solvent was reduced almost to dryness followed by addition of water, and this process was repeated four times. The product was dissolved in methanol and precipitated with petroleum ether and acetone 50:50 (v/v). The solid was washed with ethyl acetate about twenty times to eliminate the excess of ligand.

Yield: 20%. Anal. Calc for [Rh_2_(C_6_H_8_O_7_)_4_(H_2_O)_2_]: C, 28.64; H 3.2; Rh, 20.4; H_2_O, 3.5%. Found: C, 28.5; H, 3.6; Rh, 20.8; H_2_O, 4.41%. IR (KBr): ν(COOH) 1724s; ν_as_(CO_2_) 1598vs; ν_s_(CO_2_) 1411vs cm^-1^. ESI-MS (m/z) for [Rh_2_(C_6_H_7_O_7_)_4_+H]^+^: 970.8. ^13^C NMR: γ_C _(125.75 MHz, D_2_O) ppm: 46.3 (CH_2_); 76.3 (C-OH); 176.4 (CO_2_H)_β_; 179.8 (CO_2_H)_α_; 192.9 (Rh-CO_2_)_β_; 195.3 (Rh-CO_2_)_α_. UV-vis (H_2_O, nm): 586 (π*_(RhRh) _→σ*_(RhRh)_); 442 (π*_(RhRh) _→σ*_(RhO)_); 292 (σ_(RhO) _→σ*_(RhRh)_).

### • Preparation of maghemite nanoparticles functionalized with Rhodium Compound, Magh-Rh_2_(H_2_cit)_4_

Maghemite (γ-Fe_2_O_3_) nanoparticles were prepared according to procedures described previously [49]. Magnetite (Fe_3_O_4_) nanoparticles were synthesized by mixing FeCl_2 _and FeCl_3 _aqueous solutions (2:1 molar ratio) with NaOH solution under vigorous stirring. The solid was washed with distilled water until pH = 9 and oxidation of magnetite to maghemite was performed adjusting the pH to 3, stirring the dispersion under heating and constant oxygen flow. The reddish sediment was centrifuged, dispersed in water, and dialyzed for 24 hours.

In the second stage of the nanocomposite preparation procedure, the magnetic nanoparticles were functionalized with rhodium (II) citrate. For this purpose, 5 mL of the magnetic dispersion and 1 mL of rhodium (II) citrate solution (0.054 molL^-1^) were mixed and stirred for two hours at room temperature. The nanoparticles were separated by centrifugation (5000 rpm), washed three times with deionized water and thereafter dispersed in 5 mL of water. The stable magnetic solution containing Magh-Rh_2_(H_2_cit)_4 _nanoparticles was obtained by adjusting the pH to 7.

### • Preparation and characterization of Magnetoliposomes

A small unilamellar liposome based on L-α-phosphatidylcholine and L-α-lysophosphatidylcholine was made according to the modified injection method described elsewhere [28]. We used L-α-lysophosphatidylcholine because the formed vesicles are smaller and this leads to an increase in the permeability of the liposomal formulation through the cells [50]. Basically, 360 μL of an ethanolic solution containing 0.686 mM L-α-phosphatidylcholine, 0.0137 mM L-α-lysophosphatidylcholine, was injected with a syringe into 5 mL phosphate buffer solution (PBS), pH 7.4. The injection of 262 μL of maghemite nanoparticles with rhodium (II) citrate into PBS was performed at 56°C, under magnetic stirring at a flow rate 1 μL/s to a final concentration of 1.96 × 10^15 ^particle/mL.

Particle size and size distribution were obtained by laser light scattering using a particle size analyzer (Zetasizer, Malvern, UK). The magnetoliposome suspension containing the maghemite nanoparticles (Magh-Rh_2_(H_2_cit)_4_) was analyzed in a 1.0 cm quartz cell. The measurement was performed in triplicates (*n *= 3). All experiments were carried out at 25°C in the range of 100-2000 Hz.

### • Cell culture

MCF-7 human mammary carcinoma cell line (purchased from American Type Collection, ATCC, USA) and 4T1 murine mammary carcinoma cells (provided by Dr. Suzanne Ostrand-Rosenberg, Maryland, USA) were cultured in flasks (TPP, Switzerland) with Dulbecco's Modified Eagle's Medium (DMEM-Sigma, USA) containing 1% (v/v) penicillin-streptomycin (Sigma) and 10% (v/v) heat-inactivated fetal bovine serum (FBS-Gibco). Human normal breast cell line MCF-10A (donated by Dr. Maria Mitzi Brentani, USP, Brazil) was cultured with a 1:1 mixture of DMEM and F12 medium (Sigma) supplemented with 5% horse serum (Gibco), hydrocortisone (0.5 μg/mL, Sigma), insulin (1 mg/mL, Sigma), epidermal growth factor (20 ng/mL, Sigma), choleric toxin (100 ng/mL, Sigma) and 1% (v/v) penicillin-streptomycin. Cells were maintained at 37°C in humidified atmosphere with 5% CO_2_.

### • Cell treatment

Cells were seeded into 6 or 96 well culture microplates at a density of 1.4 × 10^4 ^cells/cm^2 ^and incubated for 24 h to allow cell's adhesion. Then cells were incubated with free Rh_2_(H_2_cit)_4 _(50-600 μM), Magh-Rh_2_(H_2_cit)_4_, and Lip-Magh-Rh_2_(H_2_cit)_4 _(50 μM of Rh_2_(H_2_cit)_4_) for 24, 48, and 72 h. As negative control, cells were incubated with maghemite nanoparticles and magnetoliposomes without Rh_2_(H_2_cit)_4 _at the same equimolar iron concentrations found in Magh-Rh_2_(H_2_cit)_4 _(23 mM, 3 × 10^15 ^iron particles/mL) and Lip-Magh-Rh_2_(H_2_cit)_4 _(94.5 mM, 12.5 × 10^15 ^iron particles/mL), respectively. Untreated cells correspond to the control group, while cells treated with paclitaxel, a chemotherapy widely used in clinics, represent the positive control used to validate the model cells. An equimolar dose of Rh_2_(H_2_cit)_4 _was used in the treatment of cells with paclitaxel (50 micromolar) to compare their cytotoxicity. Dimethyl sulfoxide (DMSO) was used as the paclitaxel treatment control.

### • Cell viability assay

Cell viability was estimated by MTT (Invitrogen, USA) assay. After treatment, as described above, cells were incubated with 15 μL of MTT (5 mg/mL) and 185 μL of culture medium for two and half hours at 37°C in humidified atmosphere with 5% CO_2_. Then the culture solution was removed and 200 μL of DMSO was added. The absorbance readings were performed by spectrophotometer (SpectraMax M2, Molecular Devices) using a microplate reader at a 595 nm wavelength. The relative cell viability (%) was calculated by the formula: [A]treatment/[A]control ×100, where [A]treatment is the absorbance of the tested sample and [A]control is the absorbance of control sample (containing only culture medium).

### • Cell morphology and ultra-structural analysis

The morphology and ultra-structural analysis were carried out after 48 h of treatment with free Rh_2_(H_2_cit)_4 _(50 and 500 μM). Cell morphology was visualized by AxioSkop light microscope (Zeiss, Germany) and images were captured using AxioVision (Zeiss) software. For ultra-structural analysis, cells were washed with PBS and fixed for 1 h in solution containing 2% glutaraldehyde (v/v), 2% (w/v) paraformaldeyde, and 3% (w/v) sucrose in 0.1 M sodium cacodylate buffer pH 7.2. Afterward, cells were rinsed in the same buffer and post fixed, for 40 minutes, in 1% osmium tetroxide (w/v) and 0.8% potassium ferricyanide (10 mM CaCl_2 _in 0.2 M sodium cacodylate buffer). The material was washed in distilled water and the block stained was performed for 12 h with 0.5% uranyl acetate at 4°C. Then samples were dehydrated in a graded acetone series (50-100%) for 10 minutes each and embedded in Spurr resin. Ultrathin sections were observed in a Jeol^® ^1011 transmission electron microscope (MET) at 80 kV.

### • Annexin-V/propidium iodide staining analysis

After treatments with 50 and 500 μM of free Rh_2_(H_2_cit)_4_, cells (1 × 10^6 ^cels/mL) were washed with PBS and resuspended in the solution containing 100 μL of binding buffer (10 mM of HEPES/NaOH (pH 7.4), 140 mM NaCl, 2.5 mM CaCl_2_), 5 μL of anexina-V-FITC (Biosource, USA) and propidium iodide (5 μg/mL, Invitrogen). In this step, cells were incubated for 15 minutes in the dark at room temperature. Next, 400 μL of binding buffer were added to the cells and 10,000 events for each sample were acquired by flow cytometry (Becton & Dickenson, San Jose, CA-USA). After acquisition, the analysis was done by software Cell QuestTM. Cells without staining with annexin and propidium iodide (PI) were used as negative control of fluorescence.

### • Actin filaments and nucleus staining analysis

Firstly, poly-L-lysine (1%) was added to coverslips placed in six well culture microplates and incubated overnight at 4°C. Cells were then attached to coverslips and, after 48 h of treatments with free Rh_2_(H_2_cit)_4 _(50 and 500 μM), they were washed with PBS and fixed with 3.5% paraformaldehyde for ten minutes at room temperature (RT). Next, the cells were permeabilized with 0.1% Triton-PBS for three minutes, washed with PBS, and incubated with 1% bovine serum albumin (BSA) for 30 minutes. Subsequently, the cells were stained with solution containing 2.5% Phaloidin-Alexa-Fluor 488 and 1% BSA (v/v) for 20 minutes and, after this time, 1 μg/mL of DAPI (4',6-diamidino-2-fenilindol) was added to cells for seven minutes in the dark at RT. The cells were washed twice with water, five minutes each, and then the coverslips were placed in slides with 4% N-propil galate. Afterwards, the cells were examined and images were captured by laser scanning confocal microscopy (Leica SP5). All microscopy gain and offset settings were maintained constant throughout the study.

### • Statistical Analysis

To determine the difference in the cell line's viability and in the annexin-V/propidium iodide staining among treatment groups over treatment time and cell line, an analysis of variance (ANOVA) with general linear model procedure followed by post hoc Tukey or Dunnet's test was used. Data were presented as mean value ± SEM of at least two independent experiments (SPSS, Inc., Chicago, IL, version 17.0). The IC_50 _or EC_50 _values and their 95% confidence intervals (CI 95%) were obtained by nonlinear regression (Sigma Stat; Prism 5.0; GraphPad Software Inc., San Diego, CA). The significance level was set at p < 0.05. In order to characterize the nanoparticles' size and morphology, the experimental data were fitted to a curve using a log-normal distribution function, and the modal diameter was obtained (SPSS, Inc., Chicago, IL, version 17.0).

## Competing interests

We also report that the University of Brasilia has submitted a patent application (in the Brazilian Patent Office - intellectual property number: 012110000013) to license the technology involved. The authors disclose no other potential conflicts of interest.

## Authors' contributions

MLBC was the principal investigator and takes primary responsibility for the paper. MLBC, ZGML, ARS* and SNB participated in the design of the study and SNB co-ordinated the research; MLBC, ESN, RCAP, RGSO and LHML performed the laboratory work for this study; ESN and ARS* synthesized the rhodium (II) citrate and rhodium (II) citrate-loaded nanoparticles; ARS^# ^and ACT encapsulated the rhodium (II) citrate-loaded nanoparticles in liposomes, ICRS was responsible for statistical analysis; MLBC, ESN, ARS* and ARS^# ^wrote the manuscript and all authors read and approved the final manuscript.

* Aparecido R de Souza

^# ^Andreza R Simioni

## References

[B1] Coughlin SS Ekwueme DU Breast cancer as a global health concern Cancer Epidemiol 2009 33 315 318 10.1016/j.canep.2009.10.003 19896917

[B2] Kostova I Platinum complexes as anticancer agents Recent Pat Anticancer Drug Discov 2006 1 1 22 10.2174/157489206775246458 18221023

[B3] Zhang CX Lippard SJ New metal complexes as potential therapeutics Curr Opin Chem Biol 2003 7 481 489 10.1016/S1367-5931(03)00081-4 12941423

[B4] Katsaros N Anagnostopoulou A Rhodium and its compounds as potential agents in cancer treatment Crit Rev Oncol Hematol 2002 42 297 308 10.1016/S1040-8428(01)00222-0 12050021

[B5] Junicke H Hart JR Kisko J Glebov O Kirsch IR Barton JK A rhodium (III) complex for high-affinity DNA base-pair mismatch recognition Proc Natl Acad Sci USA 2003 100 3737 3742 10.1073/pnas.0537194100 12610209 PMC152991

[B6] Angeles-Boza AM Chifotides HT Aguirre JD Chouai A Fu PK Dunbar KR Turro C Dirhodium(II, II) complexes: molecular characteristics that affect in vitro activity J Med Chem 2006 49 6841 6847 10.1021/jm060592h 17154514

[B7] Zyngier S Kimura E Najjar R Antitumor effects of rhodium (II) citrate in mice bearing Ehrlich tumors Braz J Med Biol Res 1989 22 397 401 2804473

[B8] Gupta AK Gupta M Synthesis and surface engineering of iron oxide nanoparticles for biomedical applications Biomaterials 2005 26 3995 4021 10.1016/j.biomaterials.2004.10.012 15626447

[B9] Namdeo M Saxena S Tankhiwale R Bajpai M Mohan YM Bajpai SK Magnetic nanoparticles for drug delivery applications J Nanosci Nanotechnol 2008 8 3247 3271 10.1166/jnn.2008.399 19051873

[B10] Maeda H Wu J Sawa T Matsumura Y Hori K Tumor vascular permeability and the EPR effect in macromolecular therapeutics: a review J Control Release 2000 65 271 284 10.1016/S0168-3659(99)00248-5 10699287

[B11] Asadishad B Vossoughi M Alamzadeh I In vitro release behavior and cytotoxicity of doxorubicin-loaded gold nanoparticles in cancerous cells Biotechnol Lett 2010 32 649 654 10.1007/s10529-010-0208-x 20131082

[B12] Kohler N Sun C Wang J Zhang M Methotrexate-modified superparamagnetic nanoparticles and their intracellular uptake into human cancer cells Langmuir 2005 21 8858 8864 10.1021/la0503451 16142971

[B13] Dreaden EC Mwakwari SC Sodji QH Oyelere AK El-Sayed MA Tamoxifen-poly(ethylene glycol)-thiol gold nanoparticle conjugates: enhanced potency and selective delivery for breast cancer treatment Bioconjug Chem 2009 20 2247 2253 10.1021/bc9002212 19919059 PMC2839930

[B14] Ma G Yang J Zhang L Song C Effective antitumor activity of paclitaxel-loaded poly (varepsilon-caprolactone)/pluronic F68 nanoparticles after intratumoral delivery into the murine breast cancer model Anticancer Drugs 21 261 269 10.1097/CAD.0b013e32833410a2 20016371

[B15] Kettering M Zorn H Bremer-Streck S Oehring H Zeisberger M Bergemann C Hergt R Halbhuber KJ Kaiser WA Hilger I Characterization of iron oxide nanoparticles adsorbed with cisplatin for biomedical applications Phys Med Biol 2009 54 5109 5121 10.1088/0031-9155/54/17/003 19661569

[B16] Douziech-Eyrolles L Marchais H Herve K Munnier E Souce M Linassier C Dubois P Chourpa I Nanovectors for anticancer agents based on superparamagnetic iron oxide nanoparticles Int J Nanomedicine 2007 2 541 550 18203422 PMC2676819

[B17] Sun YK Ma M Zhang Y Gu N Synthesis of nanometer-size maghemita particles from magnetite Colloids and Surfaces A: Physicochemical and Engineering Aspects 2004 245 15 19 10.1016/j.colsurfa.2004.05.009

[B18] International Center of Diffraction Data PDF Card 22-1086 2000

[B19] Batlle X Labarta A Finite-size effects in fine particles: magnetic and transport properties Journal of Physics D: Applied Physics 2002 35,:15 42 10.1088/0022-3727/35/6/201

[B20] Lu AH Salabas EL Schuth F Magnetic nanoparticles: synthesis, protection, functionalization, and application Angewandte Chemie 2007 46 1222 1244 10.1002/anie.200602866 17278160

[B21] Bellamy LJ The Infrared Spectra of Complex Molecules 1975 3 Chapman and Hall

[B22] Drmota A Kosak A Znidarsic A A mechanism for the adsorption of carboxylic acids onto the surface of magnetic nanoparticles Materials and Technology 2008 42 79 83

[B23] Boyar EB Robinson SD Rhodium(II) Carboxylates Coordination Chemistry Reviews 1983 50 109 208 10.1016/0010-8545(83)85028-0

[B24] Deng YF Jiang YQ Hong QM Zhou ZH Speciation of water-soluble titanium citrate: Synthesis, structural, spectroscopic properties and biological relevance Polyhedron 2007 26 1561 1569 10.1016/j.poly.2006.08.017

[B25] Zhou ZH Hou SY Cao ZX Tsai KR Chow YL Syntheses, spectroscopies and structures of molybdenum(VI) complexes with homocitrate Inorganic Chemistry 2006 45 8447 8451 10.1021/ic061429f 16999446

[B26] Najjar R Santos FS Seidel W Synthesis and characterization of the rhodium(II) citrate complex Anais da Academia Brasileira de Ciências 1987 59 1 2

[B27] Liu ZL Wang HB Lu QH Du GH Peng L Du YQ Zhang SM Yao KL Synthesis and characterization of ultrafine well-dispersed magnetic nanoparticles Journal of Magnetism and Magnetic Materials 2004 283 248 262

[B28] Simioni AR Pelisson MM Beltrame M Tedesco AC Photophysical and photobiological studies of a silicon tribenzonaphthoporphyrazinato incorporated into liposomes for photodynamic therapy use J Nanosci Nanotechnol 2008 8 3208 3215 10.1166/jnn.2008.108 18681070

[B29] de Souza AR Najjar R Glikmanas S Zyngier SB Water-soluble rhodium(II) carboxylate adducts: cytotoxicity of the new compounds J Inorg Biochem 1996 64 1 5 10.1016/0162-0134(95)00227-8 8837497

[B30] Howard RA Kimball AP Bear JL Mechanism of action of tetra-mu-carboxylatodirhodium(II) in L1210 tumor suspension culture Cancer Res 1979 39 2568 2573 445459

[B31] de Souza AR Coelho EP Zyngier SB Comparison of the anti-neoplastic effects of dirhodium(II) tetrapropionate and its adducts with nicotinate and isonicotinate anions in mice bearing Ehrlich tumors Eur J Med Chem 2006 41 1214 1216 10.1016/j.ejmech.2006.05.016 16822594

[B32] Chifotides HT Dunbar KR Interactions of metal-metal-bonded antitumor active complexes with DNA fragments and DNA Acc Chem Res 2005 38 146 156 10.1021/ar0302078 15709734

[B33] Gottlieb E Armour SM Harris MH Thompson CB Mitochondrial membrane potential regulates matrix configuration and cytochrome c release during apoptosis Cell Death Differ 2003 10 709 717 10.1038/sj.cdd.4401231 12761579

[B34] Mizushima N Methods for monitoring autophagy Int J Biochem Cell Biol 2004 36 2491 2502 10.1016/j.biocel.2004.02.005 15325587

[B35] Turcotte S Giaccia AJ Targeting cancer cells through autophagy for anticancer therapy Curr Opin Cell Biol 22 246 251 10.1016/j.ceb.2009.12.007 20056398 PMC4012537

[B36] Alberti C Cytoskeleton structure and dynamic behaviour: quick excursus from basic molecular mechanisms to some implications in cancer chemotherapy Eur Rev Med Pharmacol Sci 2009 13 13 21 19364082

[B37] Rosenblum MD Shivers RR 'Rings' of F-actin form around the nucleus in cultured human MCF7 adenocarcinoma cells upon exposure to both taxol and taxotere Comp Biochem Physiol C Toxicol Pharmacol 2000 125 121 131 11790336 10.1016/s0742-8413(99)00101-2

[B38] Jordan MA Wilson L Microtubules and actin filaments: dynamic targets for cancer chemotherapy Curr Opin Cell Biol 1998 10 123 130 10.1016/S0955-0674(98)80095-1 9484604

[B39] Kwok JC Richardson DR The iron metabolism of neoplastic cells: alterations that facilitate proliferation? Crit Rev Oncol Hematol 2002 42 65 78 10.1016/S1040-8428(01)00213-X 11923069

[B40] Petri-Fink A Chastellain M Juillerat-Jeanneret L Ferrari A Hofmann H Development of functionalized superparamagnetic iron oxide nanoparticles for interaction with human cancer cells Biomaterials 2005 26 2685 2694 10.1016/j.biomaterials.2004.07.023 15585272

[B41] Sinisterra RD Shastri VP Najjar R Langer R Encapsulation and release of rhodium(II) citrate and its association complex with hydroxypropyl-beta-cyclodextrin from biodegradable polymer microspheres J Pharm Sci 1999 88 574 576 10.1021/js9804314 10229652

[B42] Burgos AE Belchior JC Sinisterra RD Controlled release of rhodium (II) carboxylates and their association complexes with cyclodextrins from hydroxyapatite matrix Biomaterials 2002 23 2519 2526 10.1016/S0142-9612(01)00386-6 12033599

[B43] Lacava ZGM Azevedo RB Martins EV Lavava LM Freitas MLL Garcia VAP Biological effect of magnetic fluids: toxicity studies Journal of magnetism and magnetic materials 1999 201 431 434 10.1016/S0304-8853(99)00002-5

[B44] Morais PC Santos RL Pimenta ACM B AR D LEC Preparation and characterization of ultra-stable biocompatible magnetic fluids using citrate-coated cobalt ferrite nanoparticles Thin solid films 2006 515 266 270 10.1016/j.tsf.2005.12.079

[B45] de Freitas ER Soares PR Santos Rde P dos Santos RL da Silva JR Porfirio EP Bao SN Lima EC Morais PC Guillo LA In vitro biological activities of anionic gamma-Fe_2_O_3 _nanoparticles on human melanoma cells J Nanosci Nanotechnol 2008 8 2385 2391 10.1166/jnn.2008.275 18572653

[B46] Sahu SK Mallick SK Santra S Maiti TK Ghosh SK Pramanik P In vitro evaluation of folic acid modified carboxymethyl chitosan nanoparticles loaded with doxorubicin for targeted delivery J Mater Sci Mater Med 2010 5 1587 97 10.1007/s10856-010-3998-4 20111985

[B47] Bear JL Gray HB Rainen L Chang IM Howard R Serio G Kimball AP Interaction of Rhodium(II) carboxylates with molecules of biologic importance Cancer Chemother Rep 1975 59 611 620 1106839

[B48] Jeffery GH Basset J Mendham J Denney RC VOGEL's Textbook of quantitative chemical analysis 1989 5 New York: Longman Scientific & Technical

[B49] Kang YS Risbud S Rabolt JF Stroeve P Synthesis and characterizations of nanometer-size Fe_3_O_4 _and g-Fe_2_O_3 _particles Chemistry of Materials 1996 8 2209 2211 10.1021/cm960157j

[B50] Ralston E Blumenthal R Weinstein JN Sharrow SO Henkart P Lysophosphatidylcholine in liposomal membranes. Enhanced permeability but little effect on transfer of a water-soluble fluorescent marker into human lymphocytes Biochimica et Biophysica Acta (BBA) - Biomembranes 1980 597 543 551 10.1016/0005-2736(80)90226-6 7378402

